# Slic-Seg: A minimally interactive segmentation of the placenta from sparse and motion-corrupted fetal MRI in multiple views

**DOI:** 10.1016/j.media.2016.04.009

**Published:** 2016-12

**Authors:** Guotai Wang, Maria A. Zuluaga, Rosalind Pratt, Michael Aertsen, Tom Doel, Maria Klusmann, Anna L. David, Jan Deprest, Tom Vercauteren, Sébastien Ourselin

**Affiliations:** a Translational Imaging Group, CMIC, University College London, London, UK; b Institute for Women’s Health, University College London, London, UK; c Department of Radiology, University Hospitals KU Leuven, Leuven, Belgium; d Department of Radiology, University College London Hospital, London, UK; e Department of Obstetrics, University Hospitals KU Leuven, Leuven, Belgium

**Keywords:** Fetal MRI, Interactive method, Co-segmentation, Graph Cuts, Random forests

## Abstract

Segmentation of the placenta from fetal MRI is challenging due to sparse acquisition, inter-slice motion, and the widely varying position and shape of the placenta between pregnant women. We propose a minimally interactive framework that combines multiple volumes acquired in different views to obtain accurate segmentation of the placenta. In the first phase, a minimally interactive slice-by-slice propagation method called Slic-Seg is used to obtain an initial segmentation from a single motion-corrupted sparse volume image. It combines high-level features, online Random Forests and Conditional Random Fields, and only needs user interactions in a single slice. In the second phase, to take advantage of the complementary resolution in multiple volumes acquired in different views, we further propose a probability-based 4D Graph Cuts method to refine the initial segmentations using inter-slice and inter-image consistency. We used our minimally interactive framework to examine the placentas of 16 mid-gestation patients from MRI acquired in axial and sagittal views respectively. The results show the proposed method has 1) a good performance even in cases where sparse scribbles provided by the user lead to poor results with the competitive propagation approaches; 2) a good interactivity with low intra- and inter-operator variability; 3) higher accuracy than state-of-the-art interactive segmentation methods; and 4) an improved accuracy due to the co-segmentation based refinement, which outperforms single volume or intensity-based Graph Cuts.

## Highlights

• Minimal user interaction is needed for a good segmentation of the placenta.
• Random forests with high level features improved the segmentation.
• Higher accuracy than state-of-the-art interactive segmentation methods.
• Co-segmentation of multiple volumes outperforms single sparse volume based method.

## 1 Introduction

The placenta plays a critical role in the growth and development of the fetus during pregnancy. Placental abnormalities are a cause of poor maternal and fetal outcome. Placental attachment disorders (Mazouni et al., 2007) such as placenta accreta are due to an abnormally adherent placenta invading the myometrium, and are associated with life-threatening postpartum hemorrhage. Image-based diagnosis of placenta accreta allows for multidisciplinary planning in an attempt to minimize risks during the delivery. In monochorionic multiple pregnancy, twin-to-twin transfusion syndrome (TTTS) (Deprest et al., 2010) can result in unequal blood distribution and severe birth defects for one or both twins. Furthermore, selective intrauterine growth restriction (sIUGR) (Chalouhi et al., 2013) leads to poor growth in the twin with insufficient nourishment from the placenta. Minimally-invasive fetoscopic surgery provides an effective treatment for TTTS and sIUGR, and surgical planning (Pratt et al., 2015) can potentially reduce treatment-related morbidity and mortality. Most commonly, placental insufficiency due to poor placentation is a major cause of fetal growth restriction which can result in cerebral palsy (Spencer et al., 2014). Better placental imaging may allow prediction of placental insufficiency and targeted interventions.

**Fig. 1:**
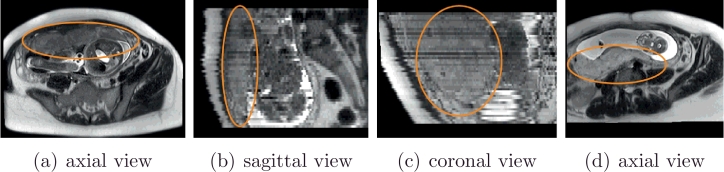
Examples of fetal MRI. (a), (b) and (c) are from one patient while (d) is from another. Note the motions and different appearance between slices in (b) and (c). The placenta is anterior in (a), but posterior in (d).

An image-based diagnosis and surgical planning system requires accurate and robust extraction of the placenta from imaging modalities with a high spatial resolution, good soft tissue contrast, and large field of view such as magnetic resonance imaging (MRI). However, high-quality 3D fetal MRI is difficult to achieve, since the free movement of the fetus can cause severe motion artifacts (Kainz et al., 2014). The Single Shot Fast Spin Echo (SSFSE) allows the motion artifacts to be nearly absent in each slice, but inter-slice motions still corrupt the volumetric data. The slices are acquired in an interleaved spatial order, which leads to inhomogeneous appearance between slices. In addition, fetal MRI is usually sparsely acquired with a large inter-slice spacing for a good contrast-to-noise ratio. Although some novel reconstruction techniques (Gholipour, Estroff, Warfield, Member, 2010, Kainz, Steinberger, Wein, Kuklisova-murgasova, Malamateniou, Keraudren, Torsney-weir, Rutherford, Aljabar, Hajnal, Rueckert, 2015) can get super-resolution volume data of fetal brain, they have yet to demonstrate their utility for placental imaging and require a dedicated non-standard acquisition protocol. Fig. 1 shows some examples of placental MRI demonstrating the current challenges for image segmentation in fetal MRI.

To address this problem, some recent methods, all dedicated to the developing fetal brain, have relied on prior knowledge learned from a group of patients or a large population to enhance the accuracy and robustness of the segmentation. For example, a shape prior model was used to extract head structures from fetal MRI (Anquez et al., 2009), and propagated atlases were used to obtain a robust segmentation of the fetal brain (Habas, Kim, Corbett-Detig, Rousseau, Glenn, Barkovich, Studholme, 2010, Gholipour, Akhondi-Asl, Estroff, Warfield, 2012). These methods assume that the variances of the target organ’s shape and position are moderate or small across different individuals. However, the position and shape of the placenta within the uterus vary greatly during gestation and between pregnancies (Fig. 1). This makes it more challenging to model such statistical prior knowledge, and also brings difficulties to automatic segmentation of the placenta.

Interactive segmentation methods have been widely used (Gao, Kikinis, Bouix, Shenton, Tannenbaum, 2012, Zhao, Xie, 2013). They provide a balance between manual delineation, which gives accurate and robust results with long segmentation time, and automatic segmentation, which saves time for user interactions but often lacks in robustness. In practical applications, an interactive segmentation method should achieve a high accuracy, minimize user interactions with a low variability among users and be computationally fast. The way in which the user inputs are used and the number of user interactions have a great impact on the segmentation accuracy. User-guided 3D active contour segmentation (Yushkevich, Piven, Hazlett, Smith, Ho, Gee, Gerig, 2006, Xu, Prince, 1998) employs the user inputs as seeds or initial contours of the target organ. Graph Cuts (Boykov and Jolly, 2001) takes user-provided scribbles as hard constraints and uses them to estimate the probabilistic model of foreground and background, which is often based on intensity distributions (Boykov, Jolly, 2001, Freedman, Zhang, 2005, Shi, Zhuang, Wolz, Simon, Tung, Wang, Ourselin, Edwards, Razavi, Rueckert, 2012). Geodesic Framework (Bai and Sapiro, 2009) and GeoS (Criminisi et al., 2008) classify a pixel based on its weighted geodesic distance to the scribbles. Random Walks (Grady et al., 2005) assigns a pixel with the label for which a random walker is most likely to reach first. GrowCut (Vezhnevets and Konouchine, 2005) uses the scribbles to set the initial state of a cellular automation for the pixel labeling task. Despite their success in many applications, most of these methods rely on low dimensional features and need a large number of user interactions to deal with images with low contrast and weak boundaries. To tackle with this problem, machine learning based methods have been proposed to learn the user intention and get an accurate segmentation with fewer user interactions (Santner, Unger, Pock, Leistner, Saffari, Bischof, 2009, Veeraraghavan, Miller, 2011, Park, Gao, Shi, Shen, 2014). For example, the 4D Active Cut proposed by Wang et al. (2014) actively selects candidate regions for querying the user, without the need to refine the segmentation slice by slice. However, its ability to deal with images with a low resolution and motion corruptions has not been investigated.

In recent years, co-segmentation methods, which combine multiple images that provide complementary information, have been demonstrated to be able to achieve better segmentation results than methods working on a single image (Guo, Yuan, Rajchl, Svenningsen, PI Capaldi, Sheikh, Fenster, Parraga, 2015, Shi, Zhuang, Wolz, Simon, Tung, Wang, Ourselin, Edwards, Razavi, Rueckert, 2012, Wang, Shi, Yap, Gilmore, Lin, Shen, 2012, Batra, Kowdle, Parikh, Luo, Chen, 2010). For fetal MRI, the high intra-slice resolution and low inter-slice resolution make it difficult to get a good segmentation result from a single 3D volume. Fortunately, fetal MRI can be acquired from different views. Although volumes acquired from different views are not completely aligned due to motion, they can be used simultaneously with their complementary resolution in different directions. Therefore, co-segmentation of fetal MRI from multiple views has the potential to provide a better accuracy and robustness.

To the best of our knowledge, there have been no previous works reported on automatic or semi-automatic segmentation of the placenta from fetal MRI. Recently we proposed a machine learning based method called Slic-Seg (Wang et al., 2015) which is designed to interactively segment the placenta from a single volume. This method minimizes the user interactions by only requiring user-provided scribbles in a single start slice. It learns from pixels that are labeled by the scribbles and infers the labels for all the remaining pixels by employing a combination of online Random Forests (RF) (Breiman, 2001) using high-level features and Conditional Random Fields (CRF) (Boykov and Jolly, 2001). Good segmentation results were achieved in our initial evaluation studies (Wang et al., 2015). However, it only worked on a single volume image, thus the performance might be negatively affected by the sparsely acquired data. In addition, its interactivity in practice and impact of high-level features and CRF were not investigated in detail.

In this paper, we extend the work of Wang et al. (2015) by using co-segmentation of multiple motion-corrupted volumes to overcome the low inter-slice resolution in a single sparsely-acquired and motion-corrupted volume. We propose a refinement step after the Slic-Seg-based single volume segmentation. The refinement takes advantage of complementary resolution in different volumes for a higher accuracy. We also validate the interactivity of Slic-Seg by analyzing how its performance is affected by the number of user interactions and measuring the operator variability.

## 2 Method

**Fig. 2:**
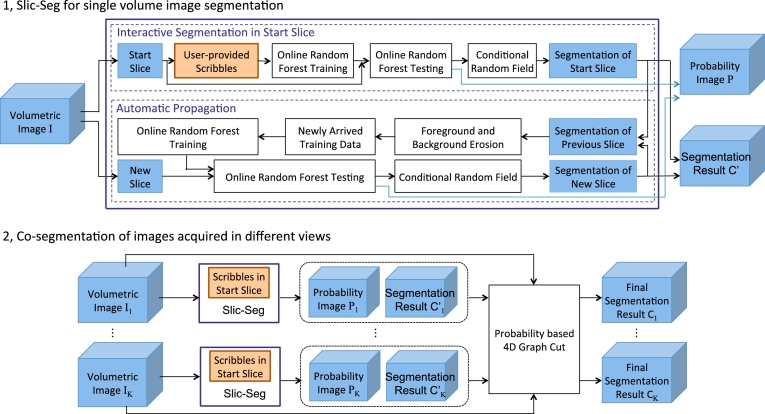
The workflow of our proposed segmentation method. In the first phase, Slic-Seg is used to segment a single volume image with minimal user interactions. In the second phase, initial segmentations of single volume Slic-Seg are refined by combining volumes acquired in different views of the same patient for an improved accuracy.

The workflow of our proposed method is shown in Fig. 2. It consists of two main phases. In the first phase, a single sparsely-acquired and motion-corrupted volume is initially segmented by single volume Slic-Seg with minimal user interactions. In the second phase, a probability-based 4D Graph Cuts framework is used to refine the initial segmentation by combining two or more volumes acquired in different views.

### 2.1 Segmentation of a single volume image with minimal user-interactions

The single volume Slic-Seg requires that a user selects a start slice and draws a few scribbles in that slice to indicate the foreground and the background. Online RF efficiently learns from these inputs and predicts the probability that an unlabeled pixel belongs to the foreground or the background. To take into account spatial consistency, that probability is incorporated into a CRF. New training data is automatically obtained from the output of the CRF and added to the training set of a RF predictor on the fly. The segmentation is propagated to other slices sequentially and automatically without the need for more user interactions. After the propagation, a volumetric probability image and an initial segmentation are obtained by stacking the output of the combined RF and CRF in all the slices respectively.

#### 2.1.1 Preprocess and feature extraction

To correct the motion between slices, a block-matching algorithm was implemented using the NiftyReg package (Ourselin et al., 2001). Feature extraction is implemented after the registration. For each pixel, features are extracted from a 9 × 9 pixel region of interest (ROI) centered on it. In each ROI, we extract gray level features including mean and standard deviation of intensity, texture features acquired by gray level co-occurrence matrix (GLCM) and wavelet coefficient features based on Haar wavelet.

#### 2.1.2 Online random forests training

A Random Forest (Breiman, 2001) is a collection of binary decision trees composed of split nodes and leaf nodes. Each tree has a maximum depth of *D*. The training set of each tree is randomly sampled from the entire labeled training set (label 1 for the placenta and label 0 for the background). At a split node, a binary test is executed to minimize the uncertainty of the class label in the subsets based on Information Gain. At a leaf node, labels of all the training samples that have been propagated to that node are averaged, and the average label is interpreted as the posterior probability of a sample belonging to the placenta, given that the sample has fallen into that leaf node.

The training data in our application is obtained in one of two ways according to the segmentation stage. For the start slice, training data comes from the scribbles provided by the user. During the propagation, after one slice *S_i_* is segmented, skeletonization of the placenta is implemented by morphological operators to get new positive training data, and the background is eroded by a kernel with a given radius (i.e., 10 pixels) to get new negative training data. The new training data obtained in *S_i_* are added to the existing training set of RF on the fly. The RF is updated and used to test the next slice $S_{i+1}$. This results in a probability map, which is combined with a CRF to get the label of $S_{i+1}$.

We use the online Bagging (Saffari et al., 2009) method to model the sequential arrival of training data as a Poisson distribution Pois(*λ*), where *λ* is set to a constant number. As each new training sample arrives, each tree is updated by choosing that sample *k* times where *k* is a random number generated by Pois(*λ*). Each sample is expected to be used *λ* times by each tree since the expectation of *k* is E(k)=λ.

#### 2.1.3 Online random forests testing

During the testing, each pixel sample **x**_*i*_ in a slice $\tilde{I}$ is propagated through all trees. For the *n*th tree, a posterior probability $p_{n}\left(c_{i}|x_{i},\tilde{I}\right)$ is obtained from the leaf that the test sample falls into, where **c**_*i*_ is the label of **x**_*i*_. The final posterior is achieved as the average across all the *N* trees.

(1) $$\begin{array}{c} p\left(c_{i}|x_{i},\tilde{I}\right)=\frac{1}{N}\sum_{n=1}^{N}p_{n}\left(c_{i}|x_{i},\tilde{I}\right) \end{array}$$

#### 2.1.4 Inference using conditional random fields

In the testing stage of RF, the posterior probability for each pixel is obtained independently, thus the result is sensitive to noise and lacks spatial consistency. To address this problem and infer the label set for all the pixels in a slice, a CRF is used for global spatial regularization. The label set $\tilde{C}$ of a slice is determined by minimizing the following energy function:

(2) $$\begin{array}{c} E\left(\tilde{C}\right)=\sum_{i\in \tilde{I}}\Psi \left(c_{i}|x_{i},\tilde{I}\right)+\lambda_{1}\sum_{\left\{i,j\right\}\in N_{1}}\Phi \left(c_{i},c_{j}|\tilde{I}\right) \end{array}$$

(3) $$\begin{array}{c} \Psi \left(c_{i}|x_{i},\tilde{I}\right)=-\log p\left(c_{i}|x_{i},\tilde{I}\right) \end{array}$$

(4) $$\begin{array}{c} \Phi \left(c_{i},c_{j}|\tilde{I}\right)=B_{i,j}\cdot \delta_{i,j} \end{array}$$

where *λ*_1_ is a coefficient to adjust the weight between two potentials. The unary potential $\Psi (c_{i}|x_{i},\tilde{I})$ measures the cost for assigning a class label **c**_*i*_ to the *i*th pixel in a slice $\tilde{I},$ and *p* comes from the output of RF. *N*_1_ is the set of all unordered pairs of {*i, j*} of neighboring pixels in the slice. The pairwise potential $\Phi (c_{i},c_{j}|\tilde{I})$ is defined as a contrast sensitive Potts model. *δ*_*i, j*_ equals to 1 if **c**_*i*_ ≠ **c**_*j*_ and 0 otherwise. *B*_*i, j*_ measures the energy due to the difference in intensity between two neighboring pixels:

(5) $$\begin{array}{c} B_{i,j}=\frac{1}{dist(i,j)}\cdot exp\left(-\frac{{\left(\tilde{I}\left(i\right)-\tilde{I}\left(j\right)\right)}^{2}}{2\sigma_{1}^{2}}\right) \end{array}$$

where $\tilde{I}\left(\cdot \right)$ denotes the intensity of one pixel. *dist*(*i, j*) is the spatial distance between two neighboring pixels, and *σ*_1_ controls the sensitivity of difference between $\tilde{I}\left(i\right)$ and $\tilde{I}\left(j\right)$. The energy minimization in Eq. (2) is solved by a max flow algorithm (Boykov and Jolly, 2001). A CRF is used in every slice of the volumetric image. After the propagation, we stack the segmentation of all slices to construct the volumetric segmentation result.

#### 2.1.5 Variations of single volume Slic-Seg

In order to analyze how each component of the above described method affects the segmentation, we consider three of its variations for comparison:

Offline Slic-Seg: this counterpart only uses user inputs in the start slice as training data for the RF. The RF is not updated when a label image is obtained for a new slice during the propagation. It uses the same high-level features and CRF as in the proposed Slic-Seg.

Slic-Seg using low-level features: this variation is the same as our proposed Slic-Seg except that it employs only intensity-based features rather than high dimensional features including GLCM and Haar wavelet.

Slic-Seg without CRF: this method uses the same high-level features and online RF as in the proposed Slic-Seg, but omits CRF. To get the binary segmentation label, the output of RF is thresholded (threshold probability is 0.5) and then the largest connected component is selected. After that morphological opening and closing operations are used to get a smoothed result.

### 2.2 Refinement based on co-segmentation of volumes acquired from different views

Since the single volume Slic-Seg implements spatial regularization by using CRF in each 2D slice, the consistency between neighboring slices is not explicitly modeled. In addition, it deals with each single volume image independently, and the large inter-slice spacing may corrupt segmentation results during the propagation. To address this problem, we refine the segmentation results of Slic-Seg by using the complementary resolution of volumes acquired from different views in a probability-based 4D Graph Cuts framework. A Fast Free-Form Deformation algorithm (Rueckert, Sonoda, Hayes, Hill, Leach, Hawkes, 1999, Modat, Ridgway, Taylor, Lehmann, Barnes, Hawkes, Fox, Ourselin, 2010) is used to register the sagittal view volume of one patient to the axial view volume of the same patient (performed at 3 levels with final grid spacing: 6 mm  ×  6 mm  ×  12 mm), but mis-alignment of placenta between them may not be perfectly addressed due to the motion and deformation. Thus, we do not impose the use of a single underlying segmentation (i.e. hard constraint) for all volumes, but rather penalize discrepancies between the segmentation of different volumes after registration(i.e, soft constraint).

Corresponding to $\tilde{I}$ and $\tilde{C}$ used in Section 2.1 to represent a 2D slice and its label respectively, we use *I* and *C*′ to represent a 3D volume image and a 3D labeling result given by single volume Slic-Seg, respectively. Considering *K* motion-corrupted volumetric images *I*_1_, *I*_2_, ... *I_K_* of the same patient sparsely acquired from different views, the user provides scribbles in a start slice of each volume respectively for the single volume Slic-Seg. The outputs of Slic-Seg for them are *P*_1_, $C_{1}^{\prime },$ *P*_2_, $C_{2}^{\prime },$ ..., *P_K_*, $C_{K}^{\prime }$ respectively, where *P_k_* denotes a probability image and each of the resulting labeled images $C_{k}^{\prime }$ is assigned with temporary values. To refine these temporary segmentations and get the final labels *C*_1_, ...,*C_K_*, Eq. (2) is extended by incorporating inter-slice and inter-image consistency:

(6) $$\begin{array}{ccc} E(C_{1},\ldots ,C_{K}) & = & \sum_{k}^{K}\sum_{i\in I_{k}}\Psi \left(c_{i}|x_{i},I_{k}\right)+\lambda_{1}\sum_{\left\{i,j\right\}\in N_{1}}B_{i,j}\cdot \delta_{i,j}\\ & & +\;\lambda_{2}\sum_{\left\{i,j\right\}\in N_{2}}B_{i,j}^{\prime }\cdot \delta_{i,j}+\lambda_{3}\sum_{\left\{i,j\right\}\in N_{3}}B_{i,j}^{\prime \prime }\cdot \delta_{i,j} \end{array}$$

where *Ψ* and *B*_*i, j*_ are defined in Eqs. (3) and (5) respectively. $B_{i,j}^{\prime }$ and $B_{i,j}^{\prime \prime }$ are the inter-slice and inter-image binary energy term, respectively. *λ*_2_ and *λ*_3_ are coefficients to adjust the weight of their corresponding terms. *N*_2_ and *N*_3_ are the set of all unordered pairs {*i, j*} of corresponding pixels from two neighboring slices and two volume images, respectively.

**Fig. 3:**
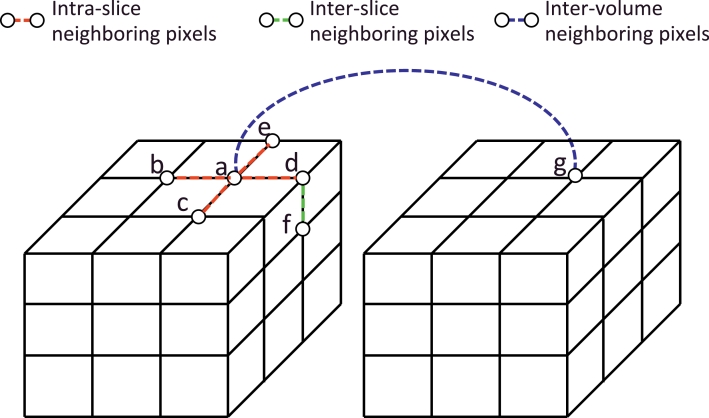
Three different kinds of neighboring pixels used in Eq. (6). {a,b}, {a,c}, {a,d}, {a,e} are intra-slice neighboring pixels (*N*_1_). {d,f} are inter-slice neighboring pixels (*N*_2_). {a,g} are inter-volume neighboring pixels (*N*_3_).

The three different types of neighboring pixels are shown in Fig. 3. {a, b}, {a, c}, {a, d} and {a, e} show intra-slice neighboring pixels that belong to *N*_1_. {d, f} shows inter-slice neighboring pixels in a single volume that belong to *N*_2_. {a, g} shows inter-volume neighboring pixels that belong to *N*_3_. To get the inter-image pixel pairs from two volumes *I*_1_ and *I*_2_, for one pixel *i* in a volume *I*_*k*1_ (k1=1,2), its nearest pixel *j* in *I*_1_ and *I*_2_ is found, and {*i, j*} is added to *N*_3_ if *j* ∈ *I*_*k*2_ (k2=1,2) and *k*1 ≠ *k*2.

To overcome the inhomogeneous appearance between different slices and between different images, the inter-slice term and inter-image term are defined based on the probability image obtained by the RF prediction in the first phase, i.e., single volume Slic-Seg:

(7) $$\begin{array}{c} B_{i,j}^{\prime }=\frac{1}{dist(i,j)}\cdot exp\left(-\frac{{\left(P_{k}\left(i\right)-P_{k}\left(j\right)\right)}^{2}}{2\sigma_{2}^{2}}\right) \end{array}$$

where $P_{k}\left(i\right)=p\left(c_{i}=1|x_{i},I_{k}\right),$ and {*i, j*} ∈ *N*_2_.

(8) $$\begin{array}{c} B_{i,j}^{\prime \prime }=exp\left(-\frac{{\left(P_{k1}\left(i\right)-P_{k2}\left(j\right)\right)}^{2}}{2\sigma_{3}^{2}}\right) \end{array}$$

where *i* ∈ *I*_*k*1_, *j* ∈ *I*_*k*2_, and {*i, j*} ∈ *N*_3_. *σ*_2_ and *σ*_3_ control the sensitivity of probability difference. Since the last term in Eq. (6) deals with corresponding pixels from different volumes, we do not use the distance between such corresponding pixels to weight the energy in Eq. (8). Instead, we set the weight to a constant value and it has been incorporated into *λ*_3_. The energy minimization problem in Eq. (6) is solved by Max flow (Boykov and Jolly, 2001), after which the final segmentation of *I*_1_, *I*_2_, ..., *I_K_* are obtained simultaneously.

## 3 Experiments and results

### 3.1 Experiment data and evaluation method

**Fig. 4:**
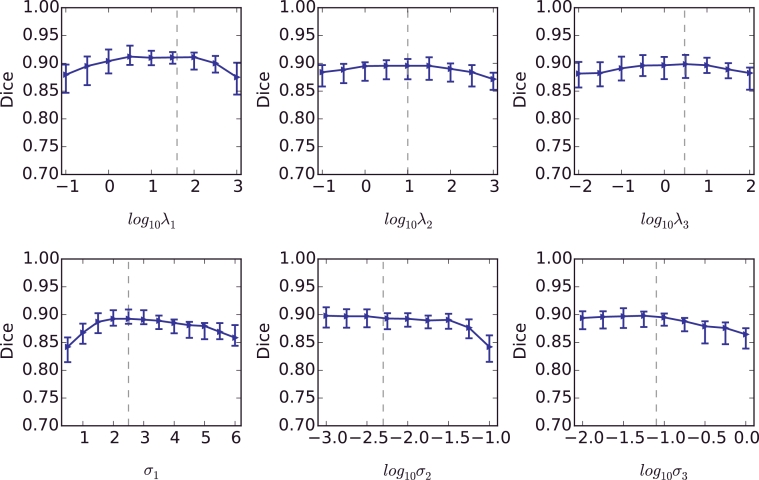
The effect of parameter change on the segmentation performance. The ranges of *λ*_1_, *λ*_2_, *λ*_3_, *σ*_2_ and *σ*_3_ are denoted by logarithms. The dashed lines indicate the parameter setting in the experiments.

We collected MRI scans of 16 fetuses in the second trimester in two different views: 1), axial view with slice dimension 512 × 448, voxel spacing 0.7422 mm × 0.7422 mm, slice thickness 3 mm. 2) sagittal view with slice dimension 256 × 256, voxel spacing 1.484 mm × 1.484 mm, slice thickness 4 mm. The slice number ranges from 50 to 70 among different volumes. For single volume Slic-Seg, a start slice in the middle region of the placenta was selected, and scribbles were provided in the start slice. The algorithm was implemented in C++ with a MATLAB GUI interface. Feature extraction was implemented in CUDA for a faster speed. The experiments were performed on a Mac laptop (OS X 10.9.5) with 16 G RAM and an Intel Core i7 CPU running at 2.5 GHz and an NVIDIA GeForce GT 750 M GPU. Parameter setting was: *λ* = 1, *D* = 10, *N* = 20, *K* = 2, *λ*_1_ = 40, *λ*_2_ = 10, *λ*_3_ = 3, *σ*_1_ = 2.5, *σ*_2_ = 0.005, *σ*_3_ = 0.08. The effect of parameter change on the segmentation performance is presented in Fig. 4, which shows stable segmentation performance was achieved with the change of each parameter over a large range.

The segmentation results were compared with manual ground truth which was annotated by an experienced radiologist. For quantitative evaluation, we measured the Dice similarity coefficient and the average symmetric surface distance (ASSD).

(9) $$\begin{array}{c} Dice=\frac{2|R_{s}\cap R_{g}|}{|R_{s}\left|+\right|R_{g}|} \end{array}$$

where $R_{s}$ and $R_{g}$ represent the region segmented by the algorithms and manual delineation of the same image, respectively.

(10) $$\begin{array}{c} ASSD=\frac{1}{|S_{s}\left|+\right|S_{g}|}\left(\sum_{i\in S_{s}}d\left(i,S_{g}\right)+\sum_{i\in S_{g}}d\left(i,S_{s}\right)\right) \end{array}$$

where $S_{s}$ and $S_{g}$ represent the set of surface points of the placenta segmented by algorithms and manual delineation respectively. $d(i,S_{g})$ is the shortest Euclidean distance between the point *i* and the surface $S_{g}$.

To evaluate the intra- and inter-user variability, we asked eight users to perform the segmentation task independently. Each user provided the scribbles for segmentation twice. The agreement between different segmentations was measured by Fleiss’ kappa coefficient (Fleiss, 1971):

(11) $$\begin{array}{c} \kappa =\frac{{\bar{P}}_{a}-{\bar{P}}_{e}}{1-{\bar{P}}_{e}} \end{array}$$

where ${\bar{P}}_{a}$ is the relative observed agreement, and ${\bar{P}}_{e}$ is the hypothetical probability of chance agreement. ${\bar{P}}_{a}$ and ${\bar{P}}_{e}$ are averaged results across all the pixels.

### 3.2 Single volume segmentation using Slic-Seg

We compared Slic-Seg with two other slice-by-slice propagation implementations which used an intensity distribution based Graph Cuts (Boykov and Jolly, 2001) (ID-GC Propagation) and a Geodesic Framework^1^ (Bai and Sapiro, 2009) (Geo-Propagation) respectively. For ID-GC, the parameter *λ* mentioned in (Boykov and Jolly, 2001) was set as 10. For Geodesic Framework, there was no parameter tuned by the user. During the propagation, they implemented the same morphological operations as in Section 2.1.2 on the obtained label of one slice to generate hard constraint for the next slice automatically. Comparisons are also made between Slic-Seg and its three variations: offline Slic-Seg, Slic-Seg using low-level features and Slic-Seg without CRF. All these methods used the same user-provided scribbles in the start slices.

#### 3.2.1 Segmentation in the start slice

**Fig. 5:**
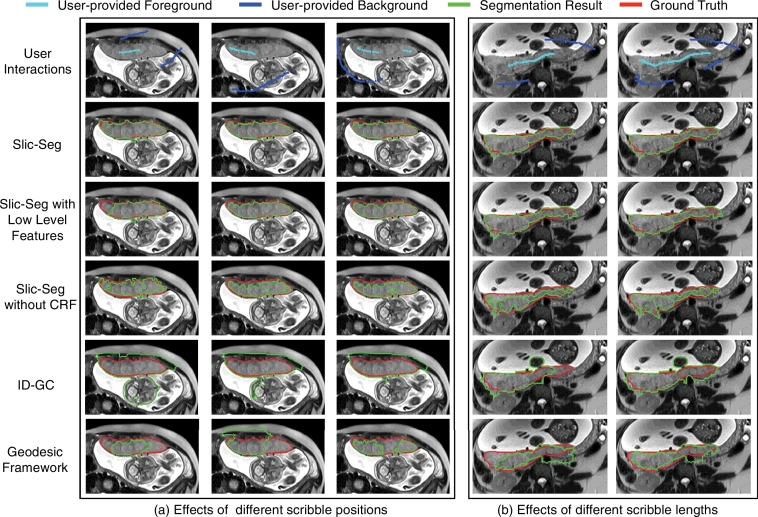
Segmentation of the placenta by different methods in the start slice. (a) shows the effects of different scribble positions. (b) shows the effects of different scribble lengths. Note the better segmentation of Slic-Seg compared to other methods.

Fig. 5 shows examples of interactive segmentation in the start slice from two patients. Since Slic-Seg and offline Slic-Seg are the same in the start slice, we omit the offline Slic-Seg here. Fig. 5(a) shows the results with different scribble positions. It can be observed that with the given scribbles, Slic-Seg has the best segmentation accuracy. In addition, it is less sensitive to the position of scribbles than other methods. Fig. 5(b) shows the effects of different scribble lengths. Scribbles in the second column are extended from that in the first column. Slic-Seg continues to provide the best accuracy. Other methods have an improved performance with the extended scribbles, but they still have some mis-segmentations, which require more user interactions to be corrected. This illustrates that Slic-Seg requires less scribbles to get good segmentation in the start slice than other compared methods.

#### 3.2.2 Segmentation during propagation

**Fig. 6:**
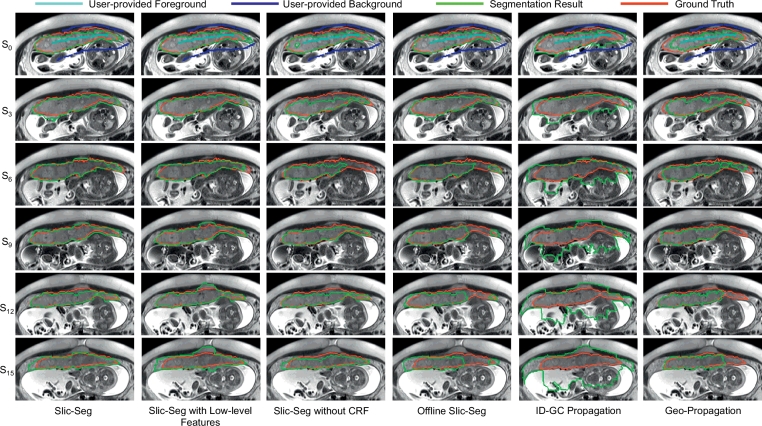
Propagation in a single volume segmentation of different methods with the same start slice and scribbles. *S_i_* represents the *i*th slice following the start slice (*S*_0_). Scribbles in *S*_0_ are extensive and all methods have a good segmentation in *S*_0_. During the propagation, only Slic-Seg keeps a high performance.

Fig. 6 shows an example of the propagation of different methods with the same user inputs(scribble length: 495 mm) in the start slice (*S*_0_). *S_i_* represents the *i*th slice following the start slice. In Fig. 6, though a good segmentation is obtained in the start slice due to an extensive set of scribbles, the errors of offline Slic-Seg, Geo-Propagation and ID-GC Propagation become increasingly large during the propagation. For Slic-Seg with low-level features, in a slice that is close to the start slice (e.g. *i* ≤ 6), it can obtain good results. When a new slice is further away (e.g. *i* ≥ 12) from the start slice, it fails to track the placenta with high accuracy. For Slic-Seg without CRF, the performance fluctuates during the propagation. In contrast, Slic-Seg has a more stable and higher performance.

**Fig. 7:**
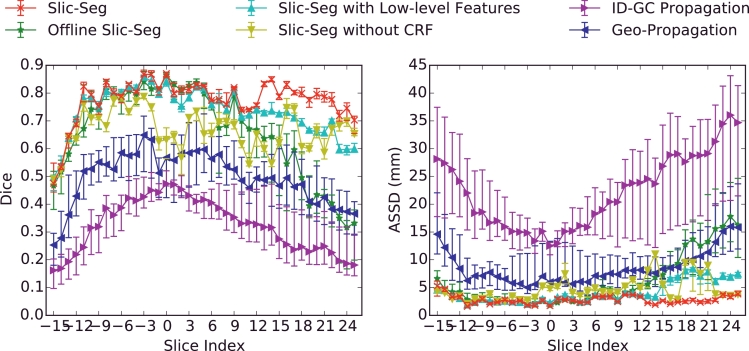
Evaluation on segmentation of a single volume with scribbles given by 8 users in terms of Dice (left) and ASSD (right) in each slice. Slice index 0 indicates the start slice.

Fig. 7 shows the Dice coefficient and ASSD for each slice in one volumetric image which was segmented by all the users. For each slice, we use error bars to show the first quartile, median and the third quartile of the Dice coefficient and ASSD. Fig. 7 shows that Slic-Seg and its variations have a better performance in the start slice and during the propagation than Geo-Propagation and ID-GC Propagation. Offline Slic-Seg and Slic-Seg with low-level features have a decreased accuracy in remote slices. The fluctuating performance of Slic-Seg without CRF is also obvious in Fig. 7. The comparison shows that Slic-Seg outperforms other methods. In addition, the lower dispersion of Slic-Seg indicates reduced variability between users.

#### 3.2.3 Interactivity and user variability

**Fig. 8:**
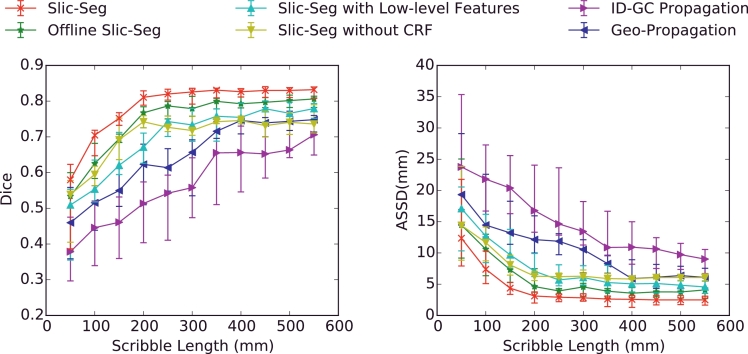
The change of Dice (left) and ASSD (right) with increasing length of scribbles that were provided in the start slice. The performance was evaluated for the segmentation of a single volume with scribbles given by 8 users.

We also measured the effects of scribble length on the accuracy for segmentation of the total volume. During the user’s drawing scribbles, the order of points on the scribbles for foreground and background was recorded, and these recorded scribbles were used sequentially and incrementally for segmentation, with the length changing from 50 mm to 550 mm. The result is shown in Fig. 8. It can be seen that Slic-Seg achieved a higher accuracy than others, with its Dice and ASSD plateauing when the length of scribbles was extended to around 200–300 mm. Fig. 8 also shows the use of online training of RF, high-level features and CRF improved the accuracy.

Since the number of slices containing the placenta varies among different volume images, we measured runtime of the propagation-based segmentation in terms of the average runtime for propagation per slice, which is defined as the ratio of the total propagation time for the volume to the number of slices containing the placenta in that volume. The time consumption by different algorithms is listed in Table 1. Note that the feature extractions for Slic-Seg and its variations are implemented on a GPU, and the propagations of all the methods are implemented on a CPU. Table 1 shows ID-GC Propagation has the smallest runtime and Slic-Seg has a larger runtime which is 1.05 ± 0.13 s per slice but still acceptable.

The mean value and standard deviation of Dice and ASSD, as well as the intra- and inter-user Fleiss’ kappa coefficient are presented in Table 2, which shows a low intra- and inter-user variability. The quantitative measurement across all the users was 0.82 ± 0.02 in terms of Dice, and 2.67 ± 0.63 mm in terms of ASSD. In addition, the intra-user *κ* ranged from 0.931 to 0.949, and the inter-user *κ* was 0.932, which indicates our interactive segmentation method has a high intra- and inter-user agreement with a low variability.

### 3.3 Refinement based on co-segmentation of multiple images

After the two volume images acquired in axial and sagittal views of one patient were segmented by single volume Slic-Seg respectively, they were co-segmented by our proposed 4D probability-based refinement (4D PR) using Graph Cuts. We compare it with three variations: 3D probability-based refinement (3D PR) using Graph Cuts, 3D intensity-based refinement (3D IR) and 4D intensity-based refinement (4D IR) using Graph Cuts. The 3D methods only consider a single volume for refinement, and the intensity-based methods define the inter-slice and inter-image binary term based on pixel intensity rather than probability.

#### 3.3.1 Refinement results

**Fig. 9:**
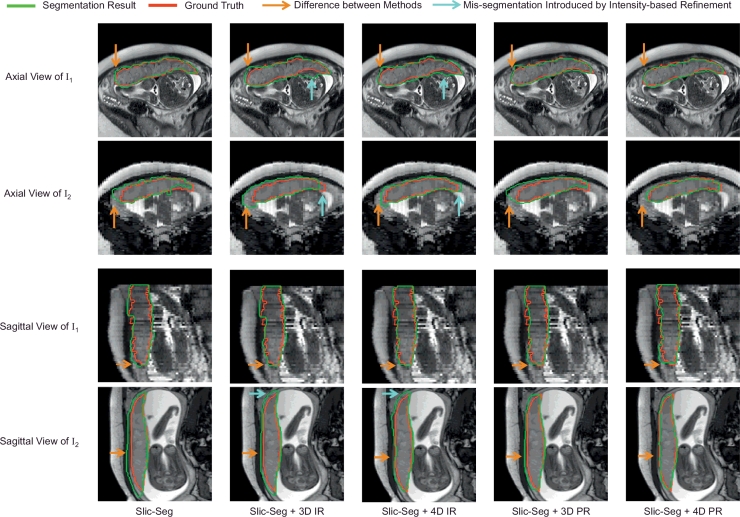
Comparison of initial segmentation by single volume Slic-Seg and refinement by 3D/4D Graph Cuts using intensity/probability respectively. *I*_1_ and *I*_2_ are acquired in two views from the same patient with complementary resolution. IR(PR) refers to intensity(probability)-based refinement.

Fig. 9 shows an example of the initial segmentation by Slic-Seg and its refined results by 3D/4D IR/PR respectively. Image *I*_1_ and *I*_2_ are acquired in two views from the same patient. *I*_1_ has a high resolution in axial view with a low resolution in sagittal view. *I*_2_ has a low resolution in axial view with a high resolution in sagittal view. The first column shows the initial segmentation of *I*_1_ and *I*_2_, both of which have some errors compared with the ground truth. The following columns show the refined segmentation results. The dark orange arrows in each row indicate the difference between the initial segmentation and the refined results. For the intensity-based methods, although some errors in the initial segmentation were corrected (the dark orange arrows in the last row), additional mis-segmentations were introduced (highlighted by the cyan arrows). Thus these two methods failed to improve the segmentation accuracy. In contrast, the probability-based methods improved the segmentation without causing extra errors. The last two columns show 4D PR outperforms 3D PR in the refinement.

We compared the above mentioned refinement methods, as well as four additional popular interactive segmentation methods for single volume segmentation: ITK-SNAP (Yushkevich et al., 2006), GeoS (Criminisi et al., 2008), 3D ID-GC (Boykov and Jolly, 2001) and GrowCut (Kikinis and Pieper, 2011). For these four methods that are not designed to accept scribbles only in a single slice, scribbles are provided in 3D, and after the segmentation the user can provide more scribbles and execute the algorithm again to correct the result. We take the results after several rounds of correction when the user confirms they are acceptable.

Quantitative evaluation are shown in Table 3, which lists the evaluation results of images acquired in axial and sagittal views respectively. The result shows Slic-Seg with 4D PR has a better performance than other interactive segmentation algorithms. In terms of the refinement, 3D IR and 4D IR achieved lower Dice values and higher ASSD values compared with the initial segmentation given by single volume Slic-Seg, which indicates that they failed to improve the segmentation accuracy. In contrast, higher accuracies than single volume Slic-Seg were achieved by the probability-based refinement methods, and 4D PR had a better performance than 3D PR. The *p* value between them is 6.9$e^{-11}$ in terms of Dice and 1.1$e^{-10}$ in terms of ASSD.

## 4 Discussion

In terms of the interactive segmentation with propagation, the experiments show that Slic-Seg achieved higher accuracy than Geodesic Framework and Graph Cuts based on intensity distributions when scribbles were given only in a single slice. The latter two methods rely on gradient or intensity information to model the placenta and background, which may not be accurate enough in fetal MRI images with poor 3D quality. Slic-Seg uses high-level features of multiple aspects including intensity, texture and wavelet coefficients. This provides a better description of the differences between the placenta and background, which is further validated by the comparison with Slic-Seg with low-level features. In addition, the online training of RF overcomes the potential appearance change when the slice-by-slice segmentation propagates to a remote slice, and the employment of CRF addresses the disconnectivity of labels resulting from RF prediction by spatial regularization. These factors allow Slic-Seg to have a good performance during the propagation. Although the use of high-level features increases the computational time, the average runtime of Slic-Seg on one slice is 1.05 s, which is acceptable for interactive segmentation. In addition, it is possible to pre-compute the features so that runtime can be reduced during the propagation. In this paper, the high-level features are designed manually, and they are not guaranteed to be the most effective features for distinguishing the placenta and the background. To improve the segmentation further, using deep learning (Sermanet, Eigen, Zhang, Mathieu, Fergus, LeCun, 2013, Roth, Lu, Farag, Shin, Liu, Turkbey, Summers, 2015) as a feature extraction method might be helpful since it can learn features automatically with large amount of training data.

The experiments show that with the increase of scribble length, better segmentations were achieved by all the compared methods, but Slic-Seg requires fewer user interactions to reach the plateau accuracy. This results in the minimization of user interactions, considering it only needs user-provided scribbles in the start slice. Besides, Table 2 shows high intra- and inter- operator agreements, which indicates a low variability within and between users.

There are three reasons to refine the segmentation results of single volume Slic-Seg in our application. First, the large inter-slice spacing and inhomogeneous appearance between slices make the accurate segmentation hard to achieve from a single volume image data. Second, Slic-Seg does not take into account the inter-slice connectivity by applying CRF only in 2D slices, which may lead to jagged surfaces in 3D space. In addition, post-segmentation refinement can be helpful considering errors in the automatic propagation. We just used the skeleton of the foreground and eroded background in one segmented slice to guide the segmentation of a following slice, which makes the error in one slice is less likely to be propagated to a following slice. As is shown in Fig. 7, the propagation of Slic-Seg is robust in most slices, and the accumulated error becomes large only in terminal slices due to a large change of the shape of the placenta between two slices. We have shown that our automatic refinement leveraging multiple volumes and relying on 4D Graph Cuts can reduce errors related to the initial propagation. To further correct the segmentations, user feedback guided refinement will be considered in future work.

The refinement method combined the complementary resolution of images acquired in different views, and reduced the segmentation errors by incorporating inter-slice and inter-image consistency. The experiment shows intensity-based 3D and 4D Graph Cuts did not improve the segmentation accuracy, indicating sole intensity information is not sufficient for good segmentation. In contrast, by defining the inter-slice and inter-image binary energy based on probability learned from RF using high-level features, large improvement of accuracy was achieved as shown in Table 3. In addition, the 4D PR achieved a better improvement in the refinement step than 3D PR, which demonstrates the co-segmentation of two images lead to higher accuracy than using a single volume image. In our current co-segmentation implementation, the *N*_3_ neighborhood is defined based on the nearest voxels from different volumes. Considering the potential alignment error, the method might be improved by defining the inter-image neighborhood based on the voxels in a local area weighted by the distance or similarity, thus mutual information or patch-based analysis (Bai et al., 2013) might be helpful for a more robust result. Note that although two images are co-segmented in the experiment, the proposed method is formulated (Eq. (6)) so that it can deal with more image volumes.

## 5 Conclusion

We presented an interactive, learning-based method for the segmentation of the placenta from motion corrupted fetal MRI in multiple views. To deal with poor image quality caused by sparse acquisition and inter-slice motion, the proposed Slic-Seg combines high-level features, Random Forests and Conditional Random Fields, which requires minimal user interactions to get good segmentation results. The segmentation was further refined by co-segmentation of images from different views using a probability-based 4D Graph Cuts method. The results demonstrated the whole segmentation framework has a good interactivity with stable performance between and within users, and large improvement of accuracy benefiting from the co-segmentation. Therefore, our approach might be suitable for segmentation of the placenta in planning systems for fetal and maternal surgery, and for rapid characterization of the placenta by MRI. Its first clinical application might be fetoscopic placement optimization in the treatment of twin-to-twin transfusion syndrome.

## Figures and Tables

**Table 1:** Average runtime per slice (in seconds) for the propagation using different methods. The feature extractions for Slic-Seg and its variations are GPU-based, and the propagations of all the methods are CPU-based.

Slic-Seg	Offline Slic-Seg	Slic-Seg with low-level features	Slic-Seg without CRF	ID-GC Propagation	Geo-Propagation
1.05 ± 0.13	0.84 ± 0.06	0.55 ± 0.10	0.93 ± 0.08	0.12 ± 0.04	0.61 ± 0.07

**Table 2:** Intra- and inter-operator variability of Slic-Seg for segmentation of volume images. *κ* is the Fleiss’s kappa coefficient in Eq. (11).

User	Dice	ASSD(mm)	*κ*
1	0.81 ± 0.02	2.73 ± 0.62	0.931
2	0.82 ± 0.03	2.57 ± 0.60	0.936
3	0.81 ± 0.03	2.75 ± 0.61	0.949
4	0.80 ± 0.03	2.81 ± 0.73	0.941
5	0.82 ± 0.02	2.58 ± 0.61	0.948
6	0.82 ± 0.02	2.63 ± 0.61	0.945
7	0.82 ± 0.02	2.61 ± 0.74	0.941
8	0.81 ± 0.03	2.76 ± 0.67	0.936
All	0.82 ± 0.02	2.67 ± 0.63	0.932

**Table 3:** Quantitative evaluation of refinement methods based on co-segmentation and comparison between popular interactive segmentation algorithms. The axial view images have a high axial-view resolution and a low sagittal-view resolution. The sagittal view images have a low axial-view resolution and a high sagittal-view resolution. The best value in each column is highlighted by bold.

Methods	Axial view			Sagittal view		
	Dice	ASSD(mm)	Time(s)	Dice	ASSD(mm)	Time(s)
ITK-SNAP	0.79 ± 0.03	2.94 ± 0.72	118.83 ± 15.35	0.81 ± 0.02	2.73 ± 0.48	106.94 ± 16.23
GeoS	0.81 ± 0.03	2.68 ± 0.67	166.72 ± 49.37	0.79 ± 0.03	3.40 ± 0.76	101.83 ± 38.84
3D ID-GC	0.79 ± 0.02	3.19 ± 0.61	188.05 ± 30.19	0.79 ± 0.03	3.57 ± 0.96	97.58 ± 10.78
Grow Cut	0.80 ± 0.03	2.78 ± 0.66	170.56 ± 23.18	0.78 ± 0.03	2.99 ± 0.85	120.38 ± 11.67
Slic-Seg	0.82 ± 0.02	2.35 ± 0.47	**81.61** **±** **17.22**	0.81 ± 0.03	2.84 ± 0.54	**47.78** **±** **13.59**
Slic-Seg + 3D IR	0.80 ± 0.03	3.28 ± 0.62	109.96 ± 21.48	0.80 ± 0.04	3.29 ± 0.72	64.84 ± 14.94
Slic-Seg + 4D IR	0.81 ± 0.03	3.00 ± 0.46	121.94 ± 23.74	0.81 ± 0.03	2.95 ± 0.58	88.11 ± 16.61
Slic-Seg + 3D PR	0.87 ± 0.03	2.16 ± 0.26	107.14 ± 23.07	0.86 ± 0.02	2.41 ± 0.45	61.82 ± 15.85
Slic-Seg + 4D PR	**0.89** **±** **0.02**	**1.89** **±** **0.39**	117.82 ± 25.53	**0.88** **±** **0.02**	**1.99** **±** **0.38**	83.98 ± 17.70

## Footnotes

1 Implementation from: http://www.robots.ox.ac.uk/~vgg/software/iseg/
